# VIST - a Variant-Information Search Tool for precision oncology

**DOI:** 10.1186/s12859-019-2958-3

**Published:** 2019-08-16

**Authors:** Jurica Ševa, David Luis Wiegandt, Julian Götze, Mario Lamping, Damian Rieke, Reinhold Schäfer, Patrick Jähnichen, Madeleine Kittner, Steffen Pallarz, Johannes Starlinger, Ulrich Keilholz, Ulf Leser

**Affiliations:** 1 Knowledge Management in Bioinformatics, Department of Computer Science, Humboldt-Universität zu Berlin, Rudower Chaussee 25, Berlin, 12489 Germany; 2 Charité Comprehensive Cancer Center, Charitéplatz 1, Berlin, 10117 Germany; 3 University Hospital Tübingen, Hoppe-Seyler-Straße 3, Tübingen, 72076 Germany; 4 Department of Hematology and Medical Oncology, Campus Benjamin Franklin, Charité Unviersitätsmedizin Berlin, Hindenburgdamm 30, Berlin, 12203 Germany; 5 Berlin Institute of Health, Kapelle-Ufer 2, Berlin, 10117 Germany; 6 German Cancer Consortium (DKTK), DKFZ Heidelberg, Im Neuenheimer Feld 280, Heidelberg, 69120 Germany

**Keywords:** Biomedical information retrieval, Document retrieval, Personalized oncology, Document classification, Clinical relevance, Document triage

## Abstract

**Background:**

Diagnosis and treatment decisions in cancer increasingly depend on a detailed analysis of the mutational status of a patient’s genome. This analysis relies on previously published information regarding the association of variations to disease progression and possible interventions. Clinicians to a large degree use biomedical search engines to obtain such information; however, the vast majority of scientific publications focus on basic science and have no direct clinical impact. We develop the Variant-Information Search Tool (VIST), a search engine designed for the targeted search of clinically relevant publications given an oncological mutation profile.

**Results:**

VIST indexes all PubMed abstracts and content from ClinicalTrials.gov. It applies advanced text mining to identify mentions of genes, variants and drugs and uses machine learning based scoring to judge the clinical relevance of indexed abstracts. Its functionality is available through a fast and intuitive web interface. We perform several evaluations, showing that VIST’s ranking is superior to that of PubMed or a pure vector space model with regard to the clinical relevance of a document’s content.

**Conclusion:**

Different user groups search repositories of scientific publications with different intentions. This diversity is not adequately reflected in the standard search engines, often leading to poor performance in specialized settings. We develop a search engine for the specific case of finding documents that are clinically relevant in the course of cancer treatment. We believe that the architecture of our engine, heavily relying on machine learning algorithms, can also act as a blueprint for search engines in other, equally specific domains. VIST is freely available at https://vist.informatik.hu-berlin.de/

**Electronic supplementary material:**

The online version of this article (10.1186/s12859-019-2958-3) contains supplementary material, which is available to authorized users.

## Background

Precision oncology denotes treatment schemes in cancer in which medical decisions depend on the individual molecular status of a patient [1]. Currently the most widely used molecular information is the patient’s genome, or, more precisely, the set of variations (mutations) an individual patient carries. Today, a number of diagnosis and treatment options already depend on the (non-)existence of certain variations in a tumor [2]. When faced with the variant profile of a patient, clinicians critically depend on accurate, up-to-date and detailed information regarding clinical implications of the present variations.

Finding such information is highly laborious and time-consuming, often taking hours or even longer for a single patient [3], as it is usually performed by manually sifting through a large volume of documents (e.g. scientific publications, clinical trial reports and case studies, among others). To find candidate documents, oncologists use search engines specialized for biomedical applications. The most popular engine, PubMed, essentially ranks search results by the date of publication [4]. Tools like GeneView [5], PubTator [6] or SemeDa [7] pre-annotate documents in their index using Named Entity Recognition (NER) to ease searching important entities like genes or drugs despite spelling variations and synonyms. They also highlight recognized entities in matching documents. DigSee [8] performs keyphrase detection for sentences describing the relationship between genes and diseases. DeepLife [9] also performs entity recognition and, in contrast to the previous tools which all consider only PubMed abstracts, also indexes certain web sites and social media content. RefMED [10] facilitates search in PubMed by user relevance feedback. However, none of these tools ranks search results according to a specific thematic focus of documents.

There are also a few search tools which are topically closer to cancer. The Cancer Hallmarks Analytics Tool [11] classifies literature based on the predefined cancer hallmarks taxonomy, but has no notion of clinical relevancy. DGIdb [12] offers search over a database of text-mined clinically relevant drug-gene pairs; in contrast, we return entire documents and have a much broader understanding of clinical relevance than just drug-gene pairs. There also exist specialized databases with manually curated evidences for variation-therapy associations, such as OncoKB [13], ClinVar [14], Clinical Interpretation of Variants in Cancer (CIViC) [15], or the Database of Curated Mutations [16]; however, these are rather small and grossly incomplete [17]. Overall, we see a clear lack of intuitive tools supporting the targeted search for clinically relevant documents in the scientific literature [18].

In this paper, we present the Variant-Information Search Tool (VIST), a search engine specifically developed to aid clinicians in precision oncology in their search for clinically relevant information for a (set of) variations or mutated genes. VIST was designed to support the inner workings of a molecular tumor board (MTB), during which a team of doctors determine the best possible cancer treatment and care plan for an individual patient. MTBs therein focus on information of direct clinical relevance, where the concept “clinical relevance” encompasses a range of different types of information, such as gene-mutation-drug associations, frequencies of variations within populations, matching clinical trials, mode of action of drugs, molecular functions and pathways associated with a variation and reports on treatments of molecularly similar tumors. Results from basic research or supported only by pre-clinical evidence is of little, if any, interest.

Besides encompassing so many different concepts, finding clinically relevant information is further complicated by the fact that central entities, such as genes, drugs, variations, or cancer entities lack a widely accepted standardized nomenclature, leading to numerous problems regarding synonyms, homonyms, and hyperonyms. To cope with these issues, VIST combines four different techniques: it (1) uses a PubMed corpus pre-annotated with state-of-the-art NER and named entity normalization tools to pre-filter documents based on genes, variations, and drug names, (2) assigns documents to different cancer entities using a classification approach, (3) mixes classical keyword search with entity search, and (4) bases its final ranking on two supervised ML classifiers trained on a silver-standard corpus obtained from two different sources. VIST furthermore offers several meta-data filters (journal, year of publication, cancer type), identifies key phrases within search results for quicker inspection [19], highlights genes, variants, drugs, and mentions of query keywords, and links out to external databases (for genes and drugs).

VIST is developed in close interaction with medical experts. We perform a number of different evaluations, including a user study with four medical experts, to assess VIST’s ranking performance. In all experiments, VIST outperforms the ranking of PubMed and of a vanilla vector space model [20] for the task of finding clinically relevant documents.

## Methods

### Architecture

**Fig. 1:**
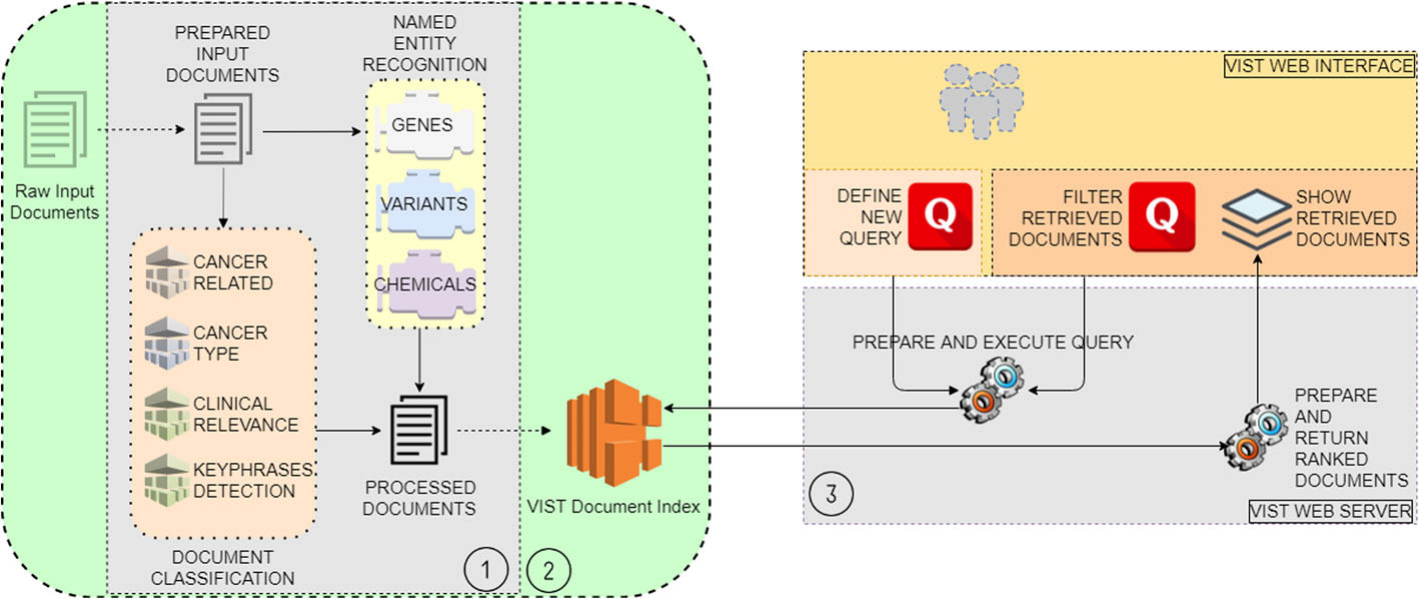
VIST System Architecture. Left: VIST backend with indexed and preprocessed documents. Right: VIST web interface for query processing and result presentation

VIST is a document retrieval system which ranks PubMed abstracts according to their clinical relevance for a (set of) variations and/or genes and a cancer entity, and also searches for relevant content in ClinicalTrials.org (CT) (which we assume as clinically relevant by default). Its architecture, presented in Fig. 1, is divided into three main components:

1. *Document Preprocessing Pipeline:* PubMed abstracts are first annotated with genes, variants, and drugs they contain. Next, pre-trained ML classification models are used to obtain query-independent relevance scores. Further classification models are used to detect key sentences with regard to oncological and clinical relevance in each individual abstract.
2. *Document Index Storage:* Built on top of Solr^1^, the document index store is used for storing annotated PubMed abstracts and CT data, and for retrieving and ranking indexed content given a user query.
3. *Web application:* The front-end user interface allows for the creation of new queries and modification of the current query. It presents matching documents ranked by clinical relevance and displays syntax-highlighted views on individual search results. The back-end of the web application parses user queries, communicates with both the Document Index Storage and the front-end, and retrieves ranked documents.

### Document preprocessing and entity annotation

PubMed documents are processed in XML-format while CT data is downloaded from the Variant Information System (VIS) for precision oncology, described in [21]. Prior to being stored in the Document Index Storage, documents undergo a comprehensive preprocessing pipeline, including textual preprocessing, meta-data extraction, document annotation, and document classification; details are described below. VIST is automatically periodically updated. This ensures that the system is populated with new content from both PubMed and CT. See Table 1 for statistics on the current VIST index (as of end of December 2018).

**Table 1:** VIST Index Summary

Property	Count
Indexed documents	29,711,223
Classified as related to cancer	630,512
Classified as clinically relevant	5,375,192
Clinically relevant & cancer	349,351
Distinct variations	433,882
Documents with >0 variations	323,722
Total number of variations	1,018,321

For annotating PubMed abstracts^2^, we first parse their XML representation using pubmed_parser^3^ [22] to extract meta-data and text (title and abstract). We then obtain entity annotation from the PubTator^4^ web service. This service detects and normalizes genes with GNormPlus [23], variations using tmVar[24], and chemicals using tmChem[25]. All three tools achieve state-of-the-art results for their respective entity types (see, for instance, [26, 27]).

### Document pre-classification

The ranking of VIST mostly depends on three query-independent scores per indexed document. These scores are obtained by classifying each document regarding a) its cancer relatedness (*CancerScore*), b) its clinical relevance (*ClinicalScore*), and c) the cancer type being discussed (*TypeScore*). The models used during these classifications are obtained by training three different classifiers on the CIViC dataset. CIViC is a cancer-oriented database of associations between human genetic variations and cancer phenotypes manually curated by medical experts. Since CIViC mostly contains documents that are related to cancer and that are clinically relevant, we added an additional negative corpus by randomly sampling 20,000 abstracts from PubMed that do not entail cancer-related terms in their title and abstract. Specifically, we used the following corpora.

**CancerScore (a):** Although the vast majority of documents in CIViC are related to cancer, there are also some which are not (*n*=68). We considered all documents with a disease annotation outside cancer as not relevant for cancer and add them to the negative corpus sampled from PubMed, treating all other documents mentioned in CIViC as positive class.

**ClinicalScore (b):** We consider each document in CIViC to be related to clinical implications of molecular lesions (n ≈ 1400) and use the randomly sampled abstracts from PubMed as negative class.

**TypeScore (c):** CIViC associates cancer types with its indexed documents. We use this information to train a multi-class classifier for the most frequent cancer types, which are melanoma, head and neck cancer, and colorectal cancer. All other cancer types are subsumed into a single class “General cancer”.

Clearly, our construction of the negative class introduces a bias into our classifiers. First, the set of negative samples and of positive samples of the first two classifiers are largely identical; only the 68 documents not related to cancer but contained in CIViC are different. Second, the ClinicalScore classifier actually will learn to discern “clinically relevant cancer document” from “non-cancer document”, instead of the more desirable “clinically relevant cancer document” from “clinically irrelevant cancer document”. However, we are not aware of any sufficiently large corpus representing the latter class. Furthermore, although the training samples are mostly identical, we observed that the models trained for the two classifiers nevertheless lead to notably different results (see Fig. 4).

For evaluating the performance of different models for the three tasks, we randomly split each data set into a training (85% of documents) and a test set (15% of documents). Statistics on the three data sets for the three classifier models are shown in Table 2. We test different classification algorithms, both neural (NN) and non-neural (non-NN) ones:

**Table 2:** Document counts of corpora used for document classification

Corpus	Size	Cancer+	Cancer-	Relevant+	Relevant-
CiVIC	1,414	1,346	68	1,414	0
PubMed	20,017	0	20,017	0	20,017

1) For the non-NN based models, we evaluate Support Vector Machine (SVM) with a linear kernel and Random Forest (RF) models, using a word n-gram representation with tf-idf weighting and *chi*^2^ for feature selection. We use the implementations available in the scikit-learn [28] package. Models are optimized by using randomized grid search for hyper-parameter optimization in a 5-fold cross-validation on the training set. We report results on the test set.

2) For NN-based models, we use two distinct approaches. First, we apply Hierarchical Attention Networks [31] (HATT), a very recent neural architecture for document classification. Additionally, we use Multi-Task Learning [29, 30] (MTL), a method which simultaneously learns different models for different yet related tasks. The novelty of this approach is that, although it eventually predicts as many results as there are tasks, it can consider correlations between these results during learning. We use HATT as the task architecture for the MTL models. In both cases, we use the pre-trained BioWordVec^5^ [31] embeddings for token representation. Most hyper parameter were left at default values. The only change we explored was the size and number of hidden layers; best results (on the training data) were obtained with 3 hidden layers of size 100 (GRU layer), 100 (Attention layer) and 50 (Dense) respectively. The architecture is the same for each of the three tasks. Classifiers are trained once on the entire training data, and we report results on the test sets.

### Document ranking

In VIST, a user query consists of a (set of) variant(s) (from a patient’s mutation profile), a (set of) gene(s), a (set of) arbitrary keyword(s), and a cancer type. Of the first three types of information, any but one may be missing; the cancer type is also optional. Queries are evaluated in the following manner. First, if a cancer type is specified, only documents classified as this type are considered. Next, if a set of variants and / or a set of genes and / or a set of keywords is specified, only documents which contain at least one of these variants or genes or keywords are considered further. All remaining documents are scored with their query-unspecific ClinicalScore and CancerScore, a query-specific KeywordScore, and the publication date. The KeywordScore is computed using a vanilla VSM as implemented in Solr. Prior to ranking, ClinicalScore and CancerScore are normalized to the interval [0;1] and multiplied to form the RankScore. The publication date is turned into a number of typecasting the year into an integer.

As for any search engine, the core of VIST is its ranking function - documents matching the query that are clinically relevant and recent should be ranked high, whereas matching documents which are of lower clinical relevance or which are older should be ranked lower. To find an appropriate ranking function, we experiment with different combinations of RankScore, CancerScore, ClinicalScore, publication date and KeywordScore as sort order, focusing on single attributes and pair-wise products. Each combination is evaluated by using the CIViC corpus as gold standard, where our hypothesis is that, for a given gene, documents in CIViC associated to this gene should be ranked high by a VIST query for this gene. To evaluate this measure, we extract all 290 genes mentioned in CIViC and extend each gene symbol with known synonyms. For each gene, we then retrieve all PubMed abstracts mentioning this gene, rank them by the score under study, and compute Mean Average Precision (MAP), Mean Reciprocal Rank (MRR) and Normalized Discounted Cumulative Gain (nDCG) of all CIViC documents in the ranked list.

### Independent evaluation sets

All evaluation data sets mentioned so far should not be considered as reliable gold standards, as they were built for tasks different from ranking by clinical relevance. We use them as silver standard corpora to fine-tune and select the classification models and ranking functions of our search engine. For assessing the performance of our final ranking function, we design three additional evaluation setups which will also be used to compare to other ranking methods or biomedical search engines. Note that none of the following data sets was used for training at any stage within our system. An overview of these corpora is given in Table 3.

**Table 3:** Overview of corpora used for evaluation

Corpus Property / Corpus	User Study	TREC PM 2017	Tumorboard
Queries	14	27	261
Documents	101	19,284	471
Unique Documents	96	16,359	325
Documents/Query	5.94	714.22	1.80
Relevant Documents	45	1,724	471
Relevant Unique Documents	44	1,681	325
Relevant/Query	3.21	63.85	1.80
Irrelevant Documents	56	17,560	-
Irrelevant Unique Documents	53	14,980	-
Irrelevant/Query	3.29	650.37	-

Properties are expressed as number of occurrences

**User study.** To obtain a set of certainly clinically (ir)relevant documents, we performed a user study encompassing four medical experts. We gathered a set of 20 queries each consisting of a gene, of a gene and a variation within this gene, or of multiple genes, as these are the typical cases occurring in recent real treatment situations at the Charité Comprehensive Cancer Center (CCCC)^6^. For each query, we used Solr VSM to find (up to) 10 matching publications. Next, each of the four experts assessed the clinical relevance (using a 5-point Likert scale) of each returned document given the query, resulting in a set of 188 triples *<Query, Document, Relevance assessment>*. To obtain a robust evaluation set, we (1) removed all pairs *<Query, Document>* which were assessed as “highly relevant” by at least one expert and as “not relevant at all” by at least one other expert and (2) obtained final assessments for all other pairs by majority voting. This results in a list of 101 *<Query, Document, Relevance assessment>* triples, consisting of 45 relevant and 56 irrelevant pairs, across 14 queries. The queries themselves are of the *<Gene(s), Mutation(s)>* format. We name this dataset *UserStudy*; it is available as Additional file 1 (AF1).

**TREC Precision Medicine.** Additionally, we use the TREC Precision Medicine 2017 dataset (*TREC PM 2017*) [32]. The collection consist of 27 queries, with 1,724 relevant and 17,560 irrelevant documents. It allows us to generate queries of format *<Gene(s), Mutation(s)>*, with both relevant and irrelevant documents included. We name this dataset *TREC PM 2017*. However, we note that the intention of VIST is not identical to that of the TREC PM task. In particular, TREC PM evaluators also used demographic information of patients to judge relevancy, information not available within VIST. Furthermore, TREC judgments are based only on a single person, while all assessments of the UserStudy set are based on four medical experts.

**Real patient cases.** Finally, we use a real-life data set generated by oncologists working at the CCCC during meetings of the Molecular Tumor Board. For each patient, these experts curated a list of relevant genes mutated in this patient and publications describing clinical implications of this variation. The data set contains 471 clinically relevant PubMed documents for 261 genes, resulting from 113 patients. It allows us to generate queries of format *<Gene(s), Mutation(s)>*. We name this dataset *Tumorboard*.

## Results

We develop VIST, an intuitive web search engine for precision oncology that aims to help oncologists to quickly find clinically relevant information given a set of variants or mutated genes of a patient. VIST is extensively evaluated to assess and optimize its performance. In the following, we first present the VIST user interface and shortly describe its functionality. Next, we present the results of a comprehensive evaluation (1) of the different models VIST uses for ranking and (2) of the performance of different ranking functions. Finally, we compare the ranking performance of VIST with that of Solr and the ranking function implemented by PubMed.

### Web interface

**Fig. 2:**
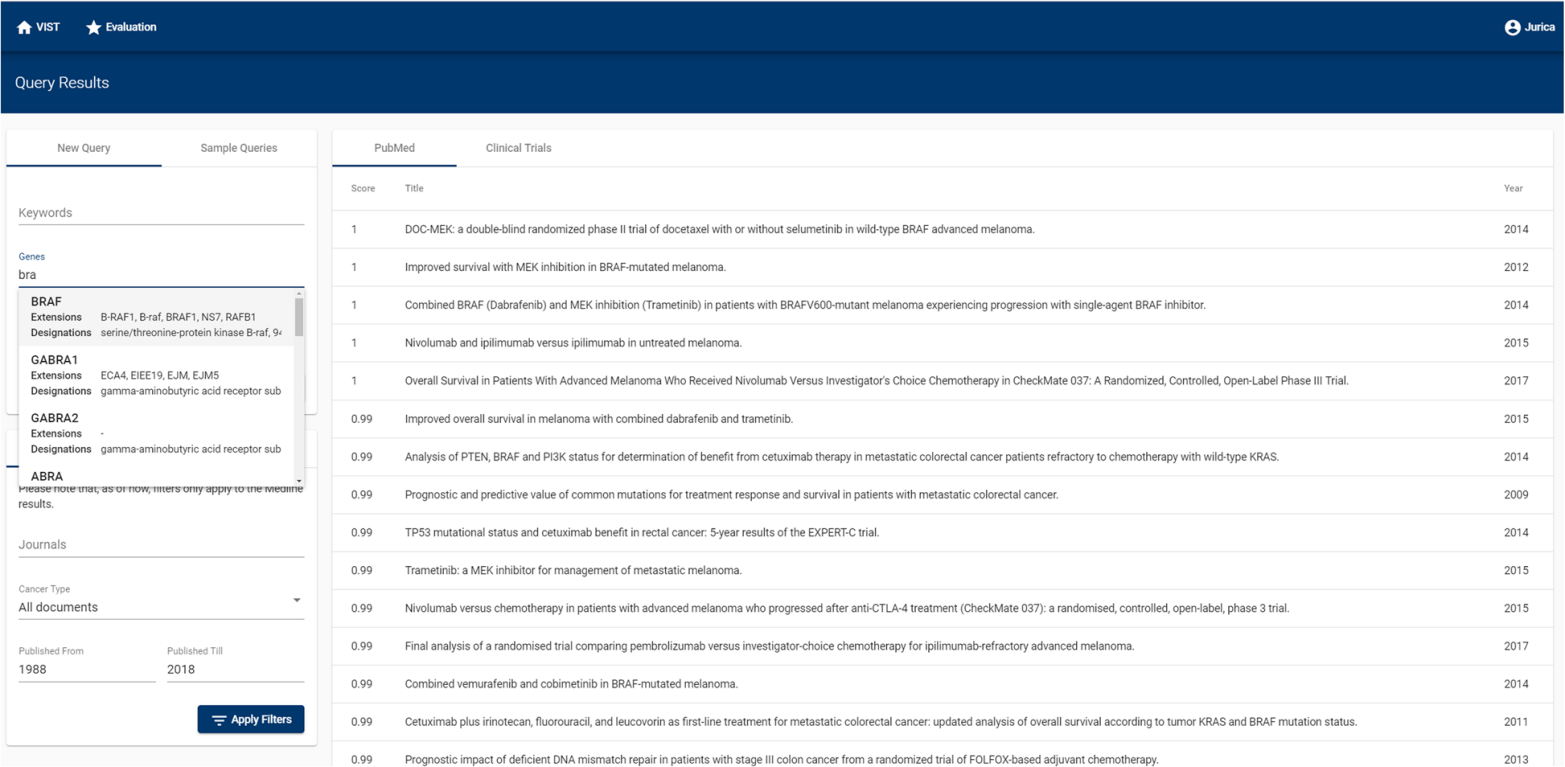
VIST web interface: Top: Search bar for entering queries. Left: Filter options (by keywords, genes, journals, cancer type, and year of publication. Main pane: List of matching documents, ranked by score according to clinical relevance. Matching clinical trials are available as a second tab

VIST’s web interface allows users to define search queries and to inspect matching documents. Additionally, it offers entity highlighting, various document filters, and a help page. The query shown in Fig. 2 is taken from the evaluation queries. It is also available in the user interface as an example query. The interface follows the principles of responsive web design.

#### Starting a new search

The initial query is of the format *Q: [Gene(s), Variant(s), Keyword(s)]*. At least one of the three items has to be specified. Keywords, genes and/or variants are used as a filter, discarding all documents which do not match the requirements. Entered gene(s) are normalized to NCBI Gene ID, with all synonyms being added to the gene query term(s). Matching abstracts are presented in a descending order based on the clinical relevance, as captured with the RankScore. For each document, its title, PMID, publication year and VIST’s RankScore are displayed. The basic interface is shown in Fig. 2. Filtering and highlighting options are enabled as soon as a search yields a non-empty result. VIST allows narrowing returned results by (a) journals, (b) year of publication, and (c) cancer type. Note that VIST presents ranked PubMed abstracts and ranked CT reports in separate tabs, as the nature of documents in these two repositories is very different, making a uniform ranking highly challenging.

#### Viewing document details

**Fig. 3:**
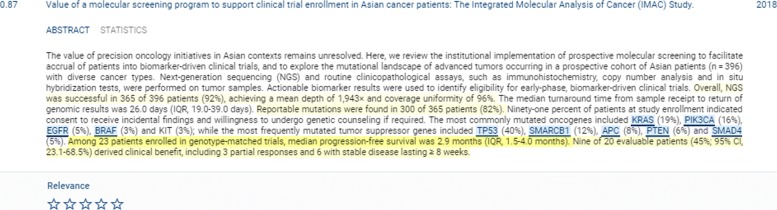
Detailed view on matching document in VIST. Entities (genes, drugs, variations) as recognized by VIST’s NER modules are highlighted. Sentences are colored according to the propbability of carrying the main message of the abstract (key phrases)

Details of a matching document can be inspected by clicking its title. Document information is provided in two tabs, *ABSTRACT* and *STATISTICS*. In the ABSTRACT tab, key sentences and annotated entities are visually highlighted (see Fig. 3). Key sentences are represented with yellow background with varying transparency levels corresponding to confidence of the detection method [19]. The STATISTICS tab shows the precomputed *ClinicalScore*, *TypeScore*, annotated variants, genes and drugs as well as MeSH keywords. It also links to the original publication. Genes and drugs are linked to relevant databases (NCBI Genes and DrugBank, respectively).

**Fig. 4:**
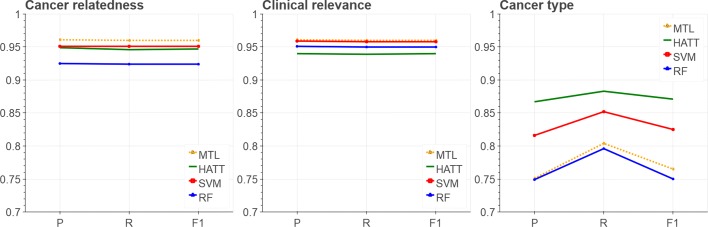
Precision (P),Recall (R) and F1 scores of three evaluated classification tasks, i.e., classification by relatedness to cancer, by clinical relevance, and by cancer type. MTL: Multi-Task Learning; HATT: Hierarchical Attention Network; SVM: Support Vector Machine; RF: Random Forest

### Query-independent classification scores

Our ranking function relies on two query-independent scores for a given document, namely its CancerScore (is this document concerned with cancer?) and its ClinicalScore (is this document concern with clinically relevant information?). In contrast, the TypeScore (which cancer entity is discussed?) is used to enable topical document filtering.

We train different classifiers for each of these tasks and compare their performance using a mixed data set of documents from CIViC and randomly sampled documents from PubMed as negative class (see Table 2). We compare both non-NN, traditional classification models and more recent, NN approaches. We do not expect the latter to clearly outperform the former, as our data sets are small compared to those where recent neural network-based methods excel [33].

P, R and F1 scores for the four types of developed classification models are shown in Fig. 4. Results for the relatively similar CancerScore and ClinicalScore are very similar among all methods, whereas the multi-class task of classifying a document by its cancer type yields more diverse and overall worse results. In the former two tasks, the MTL model is marginally better in F1-score than the second best approach, an SVM, whereas the SVM approach clearly beats MTL in the Cancer Type task. HATT performs worse than MTL for Cancer Relatedness and for Clinical Relevance, but outperforms the other methods for CancerType classification. Overall, we conclude that all four methods perform comparable, and that a definite winner cannot be identified given the deficiencies of our evaluation data, in particular the random sampling for obtaining negative documents in all three tasks. We therefore decided to further on perform experiments with only one non-NN-based model and one NN-based model. For the former, we chose SVMs as they outperform RF in all three tasks. For the latter, we chose MTL, because it performed better than HATT in two of the three tasks in Fig. 4, because MTL incorporated HATT as base classifier into its multi-task learning framework, and because the recent literature has several examples where MTL-approaches outperform other NN-models both in text-based tasks [34] and in non-text tasks [35].

### Selection of ranking function

We next evaluate different combinations of CancerScore, ClinicalScore, KeywordScore, and publication date to rank documents by their clinical relevance. To this end, we execute one query to VIST for each gene mentioned in CIViC and measure the recall of documents mentioned in CIViC for this gene among all documents indexed in VIST mentioning this gene.

Results for the three best combinations and the simple KeywordScore as baseline are shown in Table 4. The *RankScore*, specifically designed to measure clinical relevance for cancer, is included in all top performing ranking functions. However, one should keep in mind that the data set used for this evaluation is also used for training the RankScore components; thus, this result is not a surprise and cannot be considered as strong evidence for the overall quality of our ranking function; see next section for an evaluation thereof. The KeywordScore, which is completely unaware of any notion of clinical relevance but selects documents simply by the genes they contain (note that all queries here are sets of synonymous gene names), is clearly outperformed by all other functions in all evaluation metrics. Interestingly, in this evaluation the rankings based on the SVM model outperform those based on MTL in two of the three metrics, probably due to the small size of the training set we used.

**Table 4:** Best performing ranking functions

Models	SVM				MTL			
Rank by:	Recall	MAP	MRR	nDCG	Recall	MAP	MRR	nDCG
RankScorê	**0.636**	**0.113**	**0.173**	**0.307**	**0.570**	0.088	0.119	0.260
PubDate * RankScore	0.634	**0.113**	0.168	0.306	0.560	0.083	0.109	0.254
CancerScore	0.618	0.092	0.115	0.274	0.569	**0.091**	**0.121**	**0.263**
KeywordScore	0.291	0.018	0.025	0.125	0.294	0.018	0.025	0.125

All elements of a ranking function are sorted descending. The KeywordScore, completely neglecting cancer relatedness and clinical relevance of documents, is included as baseline. ^ used in production version of VIST

### Comparative evaluation

We compare the ranking of VIST with that of PubMed (using Entrez E-utilities [36], with returned documents sorted by their relevance to the query [37]) and that of a plain VSM ranking using Solr (KeywordScore). For queries containing more than one gene, we combined the resulting keywords with a logical OR in all systems. We used the three evaluation data sets *UserStudy*, *TREC PM17*, and *Tumorboard* which all are disjoint from the data sets used for training our models. Again, we primarily use the standard information retrieval metrics MAP, MRR, and nDCG. However, we also introduce a fourth metric to acknowledge the fact that VIST filters results based on variant / gene / cancer types. One could argue that this gives an undue advantage to VIST compared to its two competitors which do not apply such filtering, as the ranks of relevant documents will be generally lower due to the filtering effect. To normalize such effects, we report the *Rel VS IrRel* metric, which measures the ratio of the average position of relevant documents to the average position of irrelevant documents. For instance, if one method ranks relevant documents at positions 1, 5, and 10 and irrelevant documents at positions 3, 6, 12, then the average rank of the relevant documents would be 16/3=5.33, the average rank of the irrelevant documents would be 21/3=7, and the ratio would be 5,33/7=0.76. This would be considered a worse ranking than that of a method ranking relevant documents at positions 55, 103, and 116 (average 91.33) and irrelevant ones at 44, 201, 240 (average 161.66). A lower value for this metric thus means that relevant documents are ranked considerably better (higher) than irrelevant documents.

**Fig. 5:**
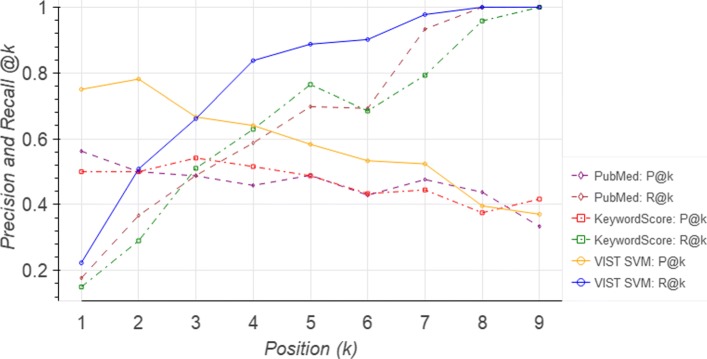
Evaluation results based on the UserStudy data set: Precision at k (P@k) and recall at k (R@k) of three different ranking schemes, i.e, PubMed, KeywordScore, and VIST SVM. Here, k refers to the k’th document in a ranked list that is also contained in the reference list

Results are shown in Table 5. VIST SVM outperforms its competitors on *TREC PM 2017* and *Tumorboard* in three out of four metrics and in all metrics on *UserStudy*. MAP, MRR, and Rel vs IrRel scores are always better that that of the PubMed ranking, MTL-based ranking, and the baseline KeywordScore. Its nDCG score is slightly worse than PubMed in *Tumorboard* and clearly worse in *TREC PM 2017*. VIST SVM is always better than VIST MTL, consistent with the results shown in Table 4. A detailed breakdown of the results for the different queries of the *UserStudy* data set reveals that VIST SVM performs best in 9 out of the 14 queries and very close to the best in the remaining five queries. VIST MTL ranks worse than the PubMed ranking for the traditional evaluation measures MAP, MRR, nDGC, but has more wins when looking at the average ranking of relevant versus irrelevant documents. Figure 5 shows average Precision@k (P@k) and Recall@k (R@k) for the three ranking approaches VIST SVM, KeywordScore, and PubMed on the *UserStudy* set; therein, k denotes the k’th document in the ranked result that is also contained in the test set. We chose this variation of the P@k and R@k metrics because the UserStudy set is rather small; ranging k over all documents returned by a method would produce precision and recall values very close to 0 for all values of k and all methods due to the construction of this corpus. The important information contained in this figure is whether or not the truly relevant ones are ranked higher than the truly irrelevant ones (according to our expert curators). Clearly, VIST outperforms KeywordScore and PubMed in both measures.

**Table 5:** Evaluation results on several datasets and several metrics

Dataset	System	MAP	MRR	nDCG	# Best Rel vs IrRel
TREC PM 2017	KeywordScore	0.0006	0.066	0.426	2
	PubMed	**0.0008**	0.056	**0.585**	5
	VIST MTL	0.0003	0.051	0.238	20*
	**VIST SVM**	**0.0008**	**0.095**	0.458	20*
Tumorboard	KeywordScore	0.0082	0.011	0.115	-
	PubMed	0.0489	0.070	**0.230**	-
	VIST MTL	0.0242	0.035	0.103	-
	**VIST SVM**	**0.0579**	**0.081**	0.220	-
UserStudy	KeywordScore	0.0631	0.296	0.645	2
	PubMed	0.0847	0.236	0.580	3
	VIST MTL	0.0571	0.239	0.407	9*
	**VIST SVM**	**0.1874**	**0.650**	**0.933**	9*

Low values are due to a small number of known PMIDs for individual queries. “# best Rel vs IrRel”: Number of queries for which the corresponding system has the best “Rel vs IrRel” score (27 queries for TREC PM 2017, 14 queries for UserStudy). *VIST SVM and VIST MTL are compared separately with KeywordScore and PubMed. KeywordScore is the ranking provided in the default settings of Solr

## Discussion

We present VIST, a specialized search engine to support the retrieval of clinically relevant literature and trial information for precision oncology, and evaluate its performance in different manners. Although our evaluation indicates that VIST ranking is superior to that of PubMed with regard to searching clinically relevant literature given mutational information, we still see a number of limitations of our current system.

Firstly, the absolute ranks of the evaluation documents in the complete result lists are typically not low; for instance, in *UserStudy*, the average rank of the first gold standard document across all queries is ≈ 150, with standard deviation ≈ 297 (≈ 230 and ≈ 325 for PubMed, respectively). This could be a problem, as the ranks might be better than in PubMed, but still not good enough for the user’s motivation to prefer VIST instead of PubMed. On the other hand, we did not evaluate the quality of the documents ranked higher than our first matches; it is very well possible that these are equally valuable as our gold standard documents. In future work, we plan to sample from these results and give them to expert evaluation.

Secondly, the current system will select and rank all documents mentioning at least one of the entities of a query, which means that the result set will grow very large for larger queries. VIST (as PubMed) has no notion of a clinically-informed prioritization of genes/variants; such a work has to be done manually prior to query formulation. Nevertheless, the ranking of VIST should rank highest those documents which contain the most clinically relevant information. Another important option we did not evaluate is the combination of variant/genes with keywords. Using such combinations, one can, for instance, easily boost the ranks of documents describing clinical trials by adding a keyword like “trial” to a query. The interplay of such user interventions with our relevance classification models remains to be studied.

Thirdly, although user feedback indicates that the integration of CT is an important feature of the system, we yet have to evaluate VIST’s performance when searching this data set. We speculate that essentially all reports in CT are of clinical relevance, thus ranking by clinical relevance makes little sense; on the other hand, not all reports will have the same importance, still calling for a proper ranking function. Currently, we only apply the KeywordScore, as all our relevance models were trained on scientific abstracts, not trial reports. Ranking within CT is thus an important topic for future work.

Fourthly, we fully acknowledge that a comprehensive investigation of variations found in a patient’s tumor must also consider other data sources, especially those containing curated information about the clinical relevance of these variations. Examples of such databases are CIViC [15], which we used for building our models, OncoKB [13], or the Precision Medicine Knowledge Base [38]. We thus see it as an important task for the community to develop tools that integrate literature search with search in multiple distributed curated knowledge bases. We recently described necessary steps into this direction in [21].

## Conclusion

We presented VIST, a novel search engine specifically designed to support patient-specific clinical investigations in precision oncology. VIST receives affected genes or individual variants as queries and produces a list of matching publications ranked according to their clinical relevance. VIST also reports matching clinical trials to help finding ongoing studies which could be relevant for the given patient. For future work, we believe that there are technical means to further improve the ranking for clinical relevance. We see the lack or sparseness of appropriate training data as the main obstacle to developing better ranking functions. One way to cope with this problem could be the usage of pre-trained latent representations of clinically relevant concepts, or the design of a better latent document representation space. For such problems, Variational AutoEncoders [39, 40] and Generative Adversarial Networks [41] recently showed promising results. Another field where recent technical advances could help is the current restriction in VIST to four cancer types. This restriction, again, is imposed by the lack of sufficient training data in CIViC for other types. Here, one could experiment with semi-supervised models, such as zero-shot learning [42, 43] or few-shot learning [44].

To address the problem of lacking gold standard corpora, VIST has a preliminary built-in module for registration of new users and subsequent user login. Note that the system can also be used without registration in a completely anonymous form. Registration is encouraged for medical professionals, as it enables giving relevance feedback. The long-term goal of this feature is 1) creation of a corpus of (ir)relevant *<User, Query, PMID, Relevance assessment>* quadruples, 2) creation of a large(r) corpus of clinically (ir)relevant scientific publications, and 3) creation of a personalized recommendation service.

## Additional file

Additional file 1 *UserStudy* queries and (ir)relevant PMID’s. (TSV 1 kb)

## Data Availability

The *UserStudy* data set is included in this published article [and its supplementary information files]. The *TREC PM 2017* relevance judgment dataset is available from http://www.trec-cds.org/qrels-treceval-abstracts.2017.txt. CiVIC is an open access database accessible from https://civicdb.org/home.

## Abbreviations

CCCC: Charitécomprehensive cancer center
CiVIC: Clinical interpretation of variants in cancer
CT: ClinicalTrials.org
HATT: Hierarchical attention networks
MAP: Mean average precision
MeSH: Medical subject headings
MRR: Mean reciprocal rank
MTB: Molecular tumor board
MTL: Multi-task learning
NCBI: National center for biotechnology information
nDCG: Normalized discounted cumulative gain
NER: Named entity recognition
NN: Neural network
P@k: Precision at k
PMID: PubMed identifier
R@k: Recall at k
Rel VS IrRel: Relevant versus irrelevant
RF: Random forest
SVM: Support vector machine
TREC: Text retrieval conference
VIS: Variant information system
VIST: Variant information search tool

## Footnotes

1
http://lucene.apache.org/solr/

2 Reports from CT currently are not entity-annotated.

3 using https://github.com/titipata/pubmed_parser

4
https://ftp://ftp.ncbi.nlm.nih.gov/pub/lu/PubTator/

5
https://github.com/ncbi-nlp/BioSentVec

6
https://cccc.charite.de/en/
